# Combined effect of ALK and MEK inhibitors in EML4–ALK-positive non-small-cell lung cancer cells

**DOI:** 10.1038/bjc.2011.586

**Published:** 2012-01-12

**Authors:** J Tanizaki, I Okamoto, K Takezawa, K Sakai, K Azuma, K Kuwata, H Yamaguchi, E Hatashita, K Nishio, P A Janne, K Nakagawa

**Affiliations:** 1 Department of Medical Oncology, Kinki University Faculty of Medicine, 377-2 Ohno-higashi, Osaka-Sayama, Osaka 589-8511, Japan; 2 Department of Genome Biology, Kinki University Faculty of Medicine, 377-2 Ohno-higashi, Osaka-Sayama, Osaka 589-8511, Japan; 3 Lowe Center for Thoracic Oncology and Department of Medical Oncology, Dana-Farber Cancer Institute, Boston, MA 02215, USA

**Keywords:** EML4–ALK, non-small-cell lung cancer, apoptosis, BIM, survivin

## Abstract

**Background:**

Although most non-small-cell lung cancer (NSCLC) patients with the echinoderm microtubule-associated protein-like 4 (*EML4*) – anaplastic lymphoma kinase (*ALK*) fusion gene – benefit from ALK tyrosine kinase inhibitors (ALK-TKIs), the efficacy of these drugs varies greatly among individuals.

**Methods:**

The antitumour action of ALK-TKIs in EML4–ALK-positive NSCLC cell lines was evaluated from their effects on cell proliferation, signal transduction, and apoptosis.

**Results:**

The ALK-TKI TAE684 inhibited cell proliferation and induced apoptosis, in association with inhibition of STAT3 and ERK phosphorylation, in EML4–ALK-positive H3122 cells. TAE684 inhibited STAT3 phosphorylation, but not ERK phosphorylation, and it showed little effect on cell proliferation or apoptosis, in EML4–ALK-positive H2228 cells. The combination of TAE684 and a MEK inhibitor-induced marked apoptosis accompanied by inhibition of STAT3 and ERK pathways in H2228 cells. Such dual interruption of STAT3 and ERK pathways induced downregulation of the antiapoptotic protein survivin and upregulation of the proapoptotic protein BIM.

**Conclusion:**

Our results indicate that interruption of both STAT3-survivin and ERK–BIM pathways is required for induction of apoptosis in NSCLC harbouring EML4–ALK, providing a rationale for combination therapy with ALK and MEK inhibitors in EML4–ALK-positive NSCLC patients for whom ALK inhibitors alone are ineffective.

Lung cancer is the leading cause of cancer deaths worldwide. Given the successful development of tyrosine kinase inhibitors (TKIs) that target the epidermal growth factor receptor (EGFR) for the treatment of individuals with lung cancer positive for EGFR mutations (Paez *et al*, 2004), the identification of other kinases implicated in lung cancer would be expected to facilitate the development of new molecularly targeted therapies. A new potential driver mutation was recently identified in 5 to 10% of cases of non-small-cell lung cancer (NSCLC): fusion of the echinoderm microtubule-associated protein-like 4 gene (*EML4*) with the anaplastic lymphoma kinase gene (*ALK*), which results in the production of a fusion protein (EML4–ALK) (Soda *et al*, 2007; Shaw *et al*, 2009; Sasaki *et al*, 2010). Early-phase clinical trials have revealed that TKIs that target ALK show marked efficacy in patients with lung cancer positive for EML4–ALK (Kwak *et al*, 2010). However, not all patients benefit from such treatment, with the clinical response to these agents thus differing even among patients who harbour this same molecular abnormality. We have now investigated possible reasons for such variability in the response to ALK-TKIs with the use of human NSCLC cell lines positive for EML4–ALK.

## Materials and methods

### Cell culture and reagents

The human NSCLC cell line H2228 was obtained from American Type Culture Collection (Manassas, VA, USA). Human NSCLC H3122 cells were obtained as previously described (Koivunen *et al*, 2008). All cells were maintained under a humidified atmosphere of 5% CO_2_ at 37 °C in RPMI 1640 medium (Sigma, St Louis, MO, USA) supplemented with 10% foetal bovine serum. TAE684, crizotinib, and AZD6244 were obtained from ShangHai Biochempartner (Shanghai, China).

### Growth inhibition assay *in vitro*

Cell viability was assessed with the MTT assay as previously described (Tanizaki *et al*, 2011).

### Annexin V binding assay

Binding of annexin V to cells was measured with the use of an Annexin-V-FLUOS Staining Kit (Roche, Basel, Switzerland), as described previously (Tanizaki *et al*, 2011).

### Immunoblot analysis

Immunoblot analysis was performed as previously described (Tanizaki *et al*, 2011). Rabbit polyclonal antibodies to human phosphorylated ALK (pY1608), to ALK, to phosphorylated signal transducer and activator of transcription 3 (STAT3), to STAT3, to phosphorylated AKT, to AKT, to poly(ADP-ribose) polymerase (PARP), and to BIM were obtained from Cell Signaling Technology (Danvers, MA, USA); those to extracellular signal-regulated kinase (ERK) and to phosphorylated ERK were from Santa Cruz Biotechnology (Santa Cruz, CA, USA); and those to *β*-actin were from Sigma.

### Gene silencing

Transfection with small interfering RNAs (siRNAs) was performed as previously described (Takezawa *et al*, 2011; Tanizaki *et al*, 2011).

### *In vivo* studies

Cubic fragments of tumour tissue (∼2 by 2 by 2 mm) were implanted subcutaneously into the axilla of 5- to 6-week-old male athymic nude mice. Treatment was initiated when tumours in each group achieved an average volume of 100–200 mm^3^. Treatment groups consisted of control, TAE684 alone, AZD6244 alone, and the combination of TAE684 and AZD6244. Each treatment group contained six mice. Each agent was administered by oral gavage daily for 26 days; control animals received a 0.5% (w/v) aqueous solution of hydroxypropylmethylcellulose as vehicle. Tumour volume was determined from caliper measurements of tumour length (*L*) and width (*W*) according to the formula *LW*^2^/2. Both tumour size and body weight were measured twice per week. Tumours were isolated at the completion of experiments, lysed, and subjected to immunoblot analysis as described above.

### Statistical analysis

Quantitative data are presented as means±s.e. and were analysed with the unpaired two-tailed Student’s *t*-test. A *P*-value of <0.05 was considered statistically significant.

## Results

### Effects of ALK-TKIs on cell proliferation and intracellular signalling in NSCLC cell lines positive for EML4–ALK

**Figure 1:**
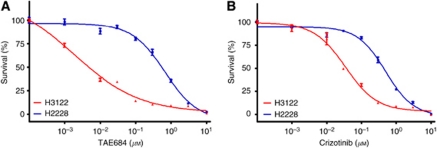
Effects of ALK inhibitors on cell proliferation in lung cancer cell lines positive for EML4–ALK. H3122 or H2228 cells were cultured for 72 h in complete culture medium-containing various concentrations of TAE684 (**A**) or crizotinib (**B**), after which cell viability was assessed. Data are expressed as percent survival and are means±s.e. of triplicates from an experiment that was repeated a total of three times with similar results.

**Figure 2:**
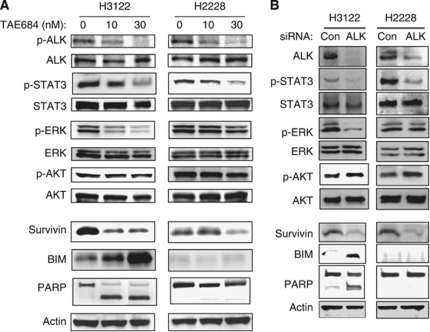
Effects of ALK inhibition on signal transduction in lung cancer cells positive for EML4–ALK. (**A**) H3122 or H2228 cells were incubated with the indicated concentrations of TAE684 for 48 h, after which cell lysates were prepared and subjected to immunoblot analysis with antibodies to phosphorylated (p) or total forms of ALK, STAT3, ERK, or AKT as well as with those to BIM, to survivin, to PARP, or to *β*-actin (loading control). The positions of the bands for EML4–ALK, BIM_EL_, and intact and cleaved forms of PARP are shown for the corresponding antibodies. (**B**) Cells were transfected with non-specific (Con) or ALK siRNAs for 48 h, after which cell lysates were prepared and subjected to immunoblot analysis as in (**A**).

We first examined the effects of the ALK-TKIs TAE684 and crizotinib on the growth of the NSCLC cell lines H3122 and H2228, both of which harbour EML4–ALK. TAE684 and crizotinib each markedly inhibited the proliferation of H3122 cells at low concentrations, whereas they affected the growth of H2228 cells only at high concentrations (Figure 1). We next evaluated the effects of TAE684 on signalling pathways in the EML4–ALK-positive NSCLC cells. Immunoblot analysis showed that phosphorylation of EML4–ALK was inhibited by TAE684 in both H3122 and H2228 cells (Figure 2A). TAE684 also markedly inhibited the phosphorylation of STAT3 and ERK as well as induced downregulation of the antiapoptotic protein survivin and upregulation of the proapoptotic protein BIM in a concentration-dependent manner in H3122 cells (Figure 2A), consistent with our previous observations (Takezawa *et al*, 2011). In addition, the generation of the cleaved form of PARP in TAE684-treated H3122 cells was indicative of the induction of apoptosis (Figure 2A). In contrast, whereas TAE684 inhibited STAT3 phosphorylation and induced downregulation of survivin in H2228 cells, it did not inhibit ERK phosphorylation or upregulate BIM at either concentration tested (Figure 2A). Furthermore, TAE684 did not induce PARP cleavage in H2228 cells (Figure 2A), indicating that it did not trigger apoptosis. To exclude the possibility that these results were due to non-specific effects of TAE684, we depleted both H3122 and H2228 cells of EML4–ALK by RNA interference (RNAi) with ALK siRNA. Depletion of EML4–ALK resulted in marked inhibition of both STAT3 and ERK phosphorylation as well as in downregulation of survivin and upregulation of BIM in H3122 cells (Figure 2B). In contrast, such depletion inhibited STAT3 phosphorylation and induced downregulation of survivin in H2228 cells, but it failed to inhibit ERK phosphorylation or to upregulate BIM (Figure 2B). Neither TAE684 nor EML4–ALK depletion had a marked effect on AKT phosphorylation in H3122 or H2228 cells (Figure 2), consistent with our previous results (Takezawa *et al*, 2011). These data thus suggested that ALK inhibitors have a pronounced antitumour effect in H3122 cells, whereas they show little such effect in H2228 cells, in spite of the fact that they inhibit the STAT3-survivin signalling pathway in these latter cells.

### Dual inhibition of STAT3 and ERK signalling pathways is required for induction of apoptosis in H2228 cells

**Figure 3:**
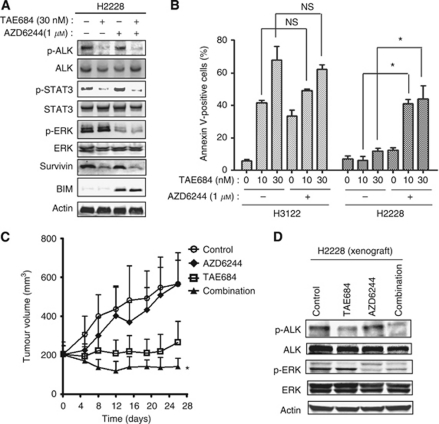
Effects of the combination of the MEK inhibitor AZD6244 with TAE684 on signal transduction and apoptosis in lung cancer cells positive for EML4–ALK. (**A**) H2228 cells were incubated in the absence or presence of TAE684 (30 nM), AZD6244 (1 *μ*M), or both agents for 48 h, after which cell lysates were prepared and subjected to immunoblot analysis with antibodies to the indicated proteins. (**B**) H3122 or H2228 cells were incubated in the absence or presence of the indicated concentrations of TAE684, AZD6244, or both agents for 60 h, after which the proportion of apoptotic cells was determined by staining with annexin V and propidium iodide followed by flow cytometry. Data are means±s.e. from three independent experiments. ^*^P<0.05 for the indicated comparisons; NS, not significant. (**C**) Nude mice with tumour xenografts established by subcutaneous implantation of H2228 cells were treated for 26 days by daily oral gavage with vehicle (control), TAE684 (0.5 mg kg^−1^), AZD6244 (25 mg kg^−1^), or the combination of both drugs. Tumour volume was determined at the indicated times after the onset of treatment. Data are means±s.e. for six mice per group. ^*^P<0.05 for the combination of TAE684 and AZD6244 *vs* either drug alone. (**D**) Lysates prepared from tumour xenografts at the completion of the experiment in (**C**) were subjected to immunoblot analysis with antibodies to the indicated proteins.

Our results suggested that sustained activation of ERK signalling in the presence of TAE684 prevented the induction of apoptosis by this drug in H2228 cells. We therefore next examined the effects of the combination of TAE684 and AZD6244, a specific inhibitor of the ERK kinase MEK, on intracellular signalling and apoptosis in H2228 cells. Immunoblot analysis revealed that AZD6244 markedly inhibited ERK phosphorylation in H2228 cells, and this effect was accompanied by upregulation of BIM (Figure 3A). Combined treatment with TAE684 and AZD6244 resulted in marked inhibition of the phosphorylation of both STAT3 and ERK as well as in both downregulation of survivin and upregulation of BIM in H2228 cells (Figure 3A). An annexin V binding assay revealed that the combination of TAE684 at 10 or 30 nM and AZD6244 induced a marked increase in the frequency of apoptosis in H2228 cells, whereas either agent alone had little such effect (Figure 3B). On the other hand, TAE684 alone induced marked apoptosis in H3122 cells, consistent with our previous results (Takezawa *et al*, 2011), and the extent of apoptosis induced by the combination of TAE684 and AZD6244 was not significantly greater than that induced by TAE684 alone (Figure 3B). Combination therapy with TAE684 and AZD6244 inhibited the growth of tumours formed by H2228 cells in nude mice to a significantly greater extent than that apparent with either drug alone (Figure 3C). We confirmed that phosphorylation of both EML4–ALK and ERK was inhibited in tumour xenografts treated with the combination of TAE684 and AZD6244, but not in those treated with TAE684 alone (Figure 3D), consistent with the results of our *in vitro* experiments. All treatments were well tolerated by the mice, with no signs of toxicity or weight loss during therapy (data not shown). These data thus suggested that additional inhibition of ERK signalling is required for the induction of apoptosis by TAE684 in H2228 cells.

### Simultaneous interruption of STAT3-survivin and ERK–BIM signalling pathways results in the induction of apoptosis in H2228 cells

**Figure 4:**
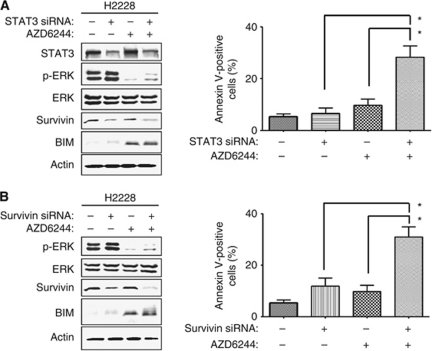
Effects of the combinations of the MEK inhibitor, AZD6244, with either STAT3 depletion or survivin depletion on signal transduction and apoptosis in lung cancer cells positive for EML4–ALK. (**A**) H2228 cells were transfected with non-specific (−) or STAT3 siRNAs and incubated with or without AZD6244 (1 *μ*M) for 48 h, after which cell lysates were subjected to immunoblot analysis with antibodies to the indicated proteins (left). Alternatively, the cells were transfected and treated with AZD6244 for 60 h, after which the proportion of apoptotic cells was determined by staining with annexin V and propidium iodide followed by flow cytometry (right). (**B**) H2228 cells were transfected with non-specific (–) or survivin siRNAs and incubated with or without AZD6244 (1 *μ*M) for 48 h, after which cell lysates were prepared and subjected to immunoblot analysis with antibodies to the indicated proteins (left). Alternatively, the cells were transfected and treated with AZD6244 for 60 h, after which the proportion of apoptotic cells was determined by staining with annexin V and propidium iodide followed by flow cytometry (right). All quantitative data are means±s.e. from three independent experiments. ^*^P<0.05 for the indicated comparisons.

To investigate whether inhibition of the STAT3-survivin pathway by TAE684 contributes to the induction of apoptosis by the combination of TAE684 and AZD6244 in H2228 cells, we depleted the cells of STAT3 by RNAi. Depletion of STAT3 resulted in the downregulation of survivin expression, and the combination of such depletion and AZD6244 treatment resulted in both downregulation of survivin and upregulation of BIM (Figure 4A). The combination of STAT3 depletion and AZD6244 treatment also resulted in a markedly greater increase in the number of apoptotic cells compared with either approach alone (Figure 4A). Similarly, the combination of survivin depletion by RNAi and AZD6244 treatment resulted in a markedly enhanced apoptotic response in H2228 cells (Figure 4B). Collectively, these data suggested that simultaneous interruption of STAT3-survivin and ERK–BIM signalling pathways is required for the induction of apoptosis in EML4–ALK-positive lung cancer cells.

## Discussion

Several TKIs that target ALK, a component of the transforming fusion protein EML4–ALK in NSCLC, have been developed (Christensen *et al*, 2007; Galkin *et al*, 2007; Soda *et al*, 2007). Although most patients with NSCLC positive for EML4–ALK derive benefit from treatment with ALK-TKIs, the clinical efficacy of these drugs varies greatly among such individuals and the molecular mechanism underlying this variability has been unclear. We have now shown that the ALK-TKIs TAE684 and crizotinib exert marked antiproliferative and proapoptotic effects in H3122 cells. In contrast, H2228 cells were resistant to the effects of these agents, consistent with previous observations that TAE684 or EML4–ALK depletion by RNAi failed to induce cell death in H2228 cells (Rikova *et al*, 2007; Koivunen *et al*, 2008).

We recently showed that the expression of BIM and that of survivin are independently regulated by ERK and STAT3 signalling pathways, respectively, and that they are implicated in ALK-TKI-induced apoptosis in NSCLC cells positive for EML4–ALK (Takezawa *et al*, 2011). Our present results show that TAE684 inhibits STAT3 phosphorylation and downregulates survivin in H2228 cells, but that it fails to inhibit ERK phosphorylation and to upregulate BIM in these cells. We found that the MEK inhibitor AZD6244 inhibits ERK phosphorylation and induces BIM expression within the clinically relevant concentration range in H2228 cells, and that the inhibition of both STAT3 and ERK pathways by the combination of TAE684 and AZD6244 was associated with a marked increase in the number of apoptotic cells. We further found that the combination of AZD6244 with depletion of either STAT3 or survivin also exhibited a pronounced proapoptotic effect in H2228 cells, supporting the notion that simultaneous interruption of STAT3 and ERK signalling pathways mediates ALK-TKI-induced apoptosis.

To investigate the mechanism responsible for the sustained activation of ERK signalling in the presence of TAE684 in H2228 cells, we sequenced full-length cDNAs derived from *KRAS*, *HRAS*, *NRAS*, *BRAF*, *CRAF*, *MEK1*, or *MEK2*; no mutations were detected in any of these genes (data not shown). We also investigated whether aberrant activation of a cell surface receptor might be responsible for this sustained ERK activation with the use of a phosphorylated receptor tyrosine kinase array, but again no such activated receptor tyrosine kinases were detected in H2228 cells (data not shown). Although the mechanism underlying the sustained activation of the ERK signalling pathway in the presence of an ALK inhibitor in H2228 cells remains unknown, our data suggest that the activity of this pathway is responsible, at least in part, for the poor response of these cells to ALK-TKIs.

In conclusion, our results suggest that interruption of both STAT3-survivin and ERK–BIM signalling is required for the induction of apoptosis in lung cancer cells harbouring EML4–ALK. Additional inhibition of the ERK pathway in the presence of TAE684 thus resulted in a pronounced antitumour effect in EML4–ALK-positive NSCLC cells that are resistant to the ALK-TKI alone. Our results thus provide a rationale for evaluation of combination therapy with ALK and MEK inhibitors in EML4–ALK-positive NSCLC patients for whom ALK inhibitors alone show little effect.

## References

[bib1] Christensen JG, Zou HY, Arango ME, Li Q, Lee JH, McDonnell SR, Yamazaki S, Alton GR, Mroczkowski B, Los G (2007) Cytoreductive antitumor activity of PF-2341066, a novel inhibitor of anaplastic lymphoma kinase and c-Met, in experimental models of anaplastic large-cell lymphoma. Mol Cancer Ther 6: 3314–3322 18089725 10.1158/1535-7163.MCT-07-0365

[bib2] Galkin AV, Melnick JS, Kim S, Hood TL, Li N, Li L, Xia G, Steensma R, Chopiuk G, Jiang J, Wan Y, Ding P, Liu Y, Sun F, Schultz PG, Gray NS, Warmuth M (2007) Identification of NVP-TAE684, a potent, selective, and efficacious inhibitor of NPM-ALK. Proc Natl Acad Sci USA 104: 270–275 17185414 10.1073/pnas.0609412103 PMC1765448

[bib3] Koivunen JP, Mermel C, Zejnullahu K, Murphy C, Lifshits E, Holmes AJ, Choi HG, Kim J, Chiang D, Thomas R, Lee J, Richards WG, Sugarbaker DJ, Ducko C, Lindeman N, Marcoux JP, Engelman JA, Gray NS, Lee C, Meyerson M, Janne PA (2008) EML4-ALK fusion gene and efficacy of an ALK kinase inhibitor in lung cancer. Clin Cancer Res 14: 4275–4283 18594010 10.1158/1078-0432.CCR-08-0168 PMC3025451

[bib4] Kwak EL, Bang YJ, Camidge DR, Shaw AT, Solomon B, Maki RG, Ou SH, Dezube BJ, Janne PA, Costa DB, Varella-Garcia M, Kim WH, Lynch TJ, Fidias P, Stubbs H, Engelman JA, Sequist LV, Tan W, Gandhi L, Mino-Kenudson M, Wei GC, Shreeve SM, Ratain MJ, Settleman J, Christensen JG, Haber DA, Wilner K, Salgia R, Shapiro GI, Clark JW, Iafrate AJ (2010) Anaplastic lymphoma kinase inhibition in non-small-cell lung cancer. N Engl J Med 363: 1693–1703 20979469 10.1056/NEJMoa1006448 PMC3014291

[bib5] Paez JG, Janne PA, Lee JC, Tracy S, Greulich H, Gabriel S, Herman P, Kaye FJ, Lindeman N, Boggon TJ, Naoki K, Sasaki H, Fujii Y, Eck MJ, Sellers WR, Johnson BE, Meyerson M (2004) EGFR mutations in lung cancer: correlation with clinical response to gefitinib therapy. Science 304: 1497–1500 15118125 10.1126/science.1099314

[bib6] Rikova K, Guo A, Zeng Q, Possemato A, Yu J, Haack H, Nardone J, Lee K, Reeves C, Li Y, Hu Y, Tan Z, Stokes M, Sullivan L, Mitchell J, Wetzel R, Macneill J, Ren JM, Yuan J, Bakalarski CE, Villen J, Kornhauser JM, Smith B, Li D, Zhou X, Gygi SP, Gu TL, Polakiewicz RD, Rush J, Comb MJ (2007) Global survey of phosphotyrosine signaling identifies oncogenic kinases in lung cancer. Cell 131: 1190–1203 18083107 10.1016/j.cell.2007.11.025

[bib7] Sasaki T, Rodig SJ, Chirieac LR, Janne PA (2010) The biology and treatment of EML4-ALK non-small cell lung cancer. Eur J Cancer 46: 1773–1780 20418096 10.1016/j.ejca.2010.04.002 PMC2888755

[bib8] Shaw AT, Yeap BY, Mino-Kenudson M, Digumarthy SR, Costa DB, Heist RS, Solomon B, Stubbs H, Admane S, McDermott U, Settleman J, Kobayashi S, Mark EJ, Rodig SJ, Chirieac LR, Kwak EL, Lynch TJ, Iafrate AJ (2009) Clinical features and outcome of patients with non-small-cell lung cancer who harbor EML4-ALK. J Clin Oncol 27: 4247–4253 19667264 10.1200/JCO.2009.22.6993 PMC2744268

[bib9] Soda M, Choi YL, Enomoto M, Takada S, Yamashita Y, Ishikawa S, Fujiwara S, Watanabe H, Kurashina K, Hatanaka H, Bando M, Ohno S, Ishikawa Y, Aburatani H, Niki T, Sohara Y, Sugiyama Y, Mano H (2007) Identification of the transforming EML4-ALK fusion gene in non-small-cell lung cancer. Nature 448: 561–566 17625570 10.1038/nature05945

[bib10] Takezawa K, Okamoto I, Nishio K, Janne PA, Nakagawa K (2011) Role of ERK-BIM and STAT3-survivin signaling pathways in ALK inhibitor-induced apoptosis in EML4-ALK-positive lung cancer. Clin Cancer Res 17: 2140–2148 21415216 10.1158/1078-0432.CCR-10-2798

[bib11] Tanizaki J, Okamoto I, Fumita S, Okamoto W, Nishio K, Nakagawa K (2011) Roles of BIM induction and survivin downregulation in lapatinib-induced apoptosis in breast cancer cells with HER2 amplification. Oncogene 29: 4097–4106 10.1038/onc.2011.111 21499301

